# 2-[3-(4-Chloro­phen­yl)-5-(4-fluoro­phen­yl)-4,5-di­hydro-1*H*-pyrazol-1-yl]-5-[(4-fluoro­phen­yl)diazen­yl]-4-methyl­thia­zole

**DOI:** 10.1107/S2414314620007002

**Published:** 2020-05-29

**Authors:** Saud A. Alanazi, Bakr F. Abdel-Wahab, Amany S. Hegazy, Benson M. Kariuki, Gamal A. El-Hiti

**Affiliations:** a Cornea Research Chair, Department of Optometry, College of Applied Medical Sciences, King Saud University, PO Box 10219, Riyadh 11433, Saudi Arabia; b Applied Organic Chemistry Department, National Research Centre, Dokki, Giza 12622, Egypt; c School of Chemistry, Cardiff University, Main Building, Park Place, Cardiff CF10 3AT, Wales; Purdue University, USA

**Keywords:** crystal structure, pyrazole, thia­zole, heterocycles

## Abstract

The mol­ecule of the title compound, C_25_H_18_ClF_2_N_5_S, comprises almost co-planar fluoro­phenyl, methyl­thia­zolyl, pyrazolyl and chloro­phenyl rings with the second fluoro­phenyl ring almost perpendicular to this plane. One fluoro­phenyl group is disordered over two components of occupancy ratio 0.767 (10):0.233 (10) related by a 24.2 (8)° twist. In the crystal, two mol­ecules related by inversion symmetry are linked by a pair of C—H⋯F contacts in an *R*(8)_2_^2^ geometry.

## Abstract 2

Pairs of mol­ecules related by inversion symmetry are linked by inter­molecular C—H⋯F contacts with *R*(8)_2_^2^ geometry.

## Graphical abstract

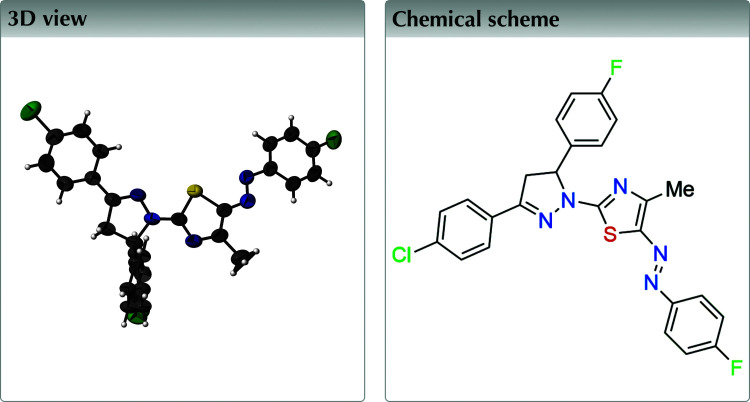

## Structure description

Various pyrazolinyl thia­zoles have pharmacological and biological applications (Abdel-Wahab *et al.*, 2017 ▸; Abd-Rabou *et al.*, 2018 ▸; Saeed *et al.*, 2017 ▸). In addition, heterocycles containing both pyrazole and thia­zole moieties have been used as versatile inter­mediates in organic synthesis of biologically active compounds (Secrieru *et al.*, 2019 ▸; Shaabani *et al.*, 2019 ▸; Sharma *et al.*, 2020 ▸). Recently, we have published the X-ray crystal structures for related heterocycles (El-Hiti, Abdel-Wahab, Alqahtani *et al.*, 2019 ▸; El-Hiti, Abdel-Wahab, Yousif *et al.*, 2019 ▸; El-Hiti *et al.*, 2018 ▸).

**Figure 1:**
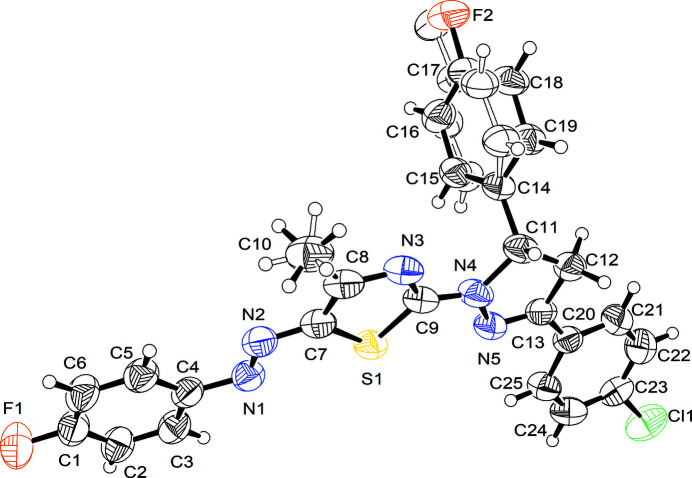
*ORTEP* representation of the title mol­ecule showing 50% probability ellipsoids.

The mol­ecule of the title compound (Fig. 1 ▸) includes fluoro­phenyl (*A*, F1/C1–C6), methyl­thia­zolyl (*B*, S1/N3,C7–C18), pyrazolyl (*C*, N4/N5/C11–C13), chloro­phenyl (*D*, Cl1/C20–C25) and fluoro­phenyl (*E*, F2/C14–C19) rings. Fluoro­phenyl group *E* is disordered over two components with an occupancy ratio of 0.767 (10):0.233 (10) and related by a twist of 24.2 (8)°.

Rings *A*–*D* are close to coplanar with twist angles *A*/*B*, *B*/*C* and *C*/*D* of 4.76 (10)°, 6.51 (11)° and 10.46 (11)° respectively. Ring *E* is almost perpendicular to *A*–*D* with a *C*/*E* twist angle of 72.66 (3)° for the major component of *E*.

**Figure 2:**
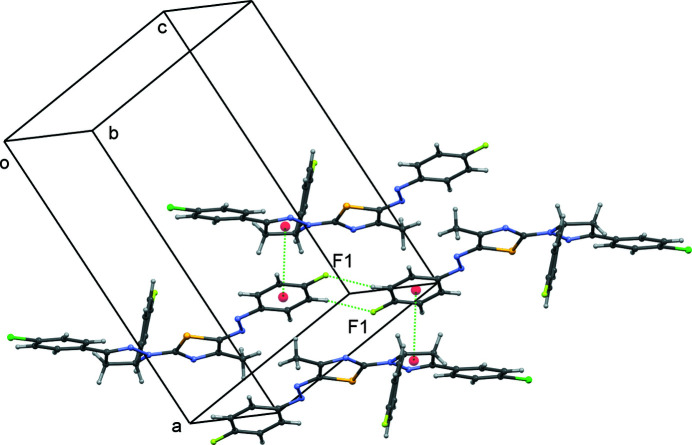
A segment of the crystal structure showing inter­molecular contacts for the major component of the disordered structure.

In the crystal structure, two mol­ecules related by inversion symmetry are linked by a pair of C—H⋯F contacts (Table 1 ▸, Fig. 2 ▸) with *R*(8)_2_^2^ geometry to form a dimer. The pyrazolyl and fluoro­phenyl rings of neighbouring mol­ecules are almost parallel with a centroid-to-centroid distance of 3.6510 (13) Å.

## Synthesis and crystallization

A mixture of 3-(4-chloro­phen­yl)-5-(4-fluoro­phen­yl)-4,5-di­hydro-1*H*-pyrazole-1-carbo­thio­amide (0.67 g, 2.0 mmol), *N′*-(4-fluoro­phen­yl)-2-oxo­propane­hydrazonoyl bromide (0.52 g, 2.0 mmol), and tri­ethyl­amine (0.20 g, 2.0 mmol) in anhydrous ethanol (20 ml) was stirred for 2 h under reflux. The solid obtained on cooling was collected by filtration, washed with ethanol, dried and recrystallized from di­methyl­formamide solution to give colourless crystals of the title compound in 86% yield (0.85 g; 1.7 mmol), m.p. 243°C, IR (KBr; cm^−1^): 1590 (N=N), 1625 (C=C), 1650 (C=N).

## Refinement

Crystal data, data collection and structure refinement details are summarized in Table 2 ▸. One fluoro­phenyl group is disordered. The two components were restrained to have similar geometries as the other ordered fluoro­phenyl group (SAME command of *SHELXL*, e.s.d. = 0.01 and 0.02 Å) and *U* ^ij^ components of disordered atoms’ ADPs were restrained to be similar to each other if within 2.0 Å distance (SIMU restraint of *SHELXL*, e.s.d. = 0.01 Å^2^). Refinement gave an occupancy ratio of 0.767 (10):0.233 (10) for the two components related by a twist of 24.2 (8)°.

## Supplementary Material

Crystal structure: contains datablock(s) I. DOI: 10.1107/S2414314620007002/zl4041sup1.cif

Structure factors: contains datablock(s) I. DOI: 10.1107/S2414314620007002/zl4041Isup2.hkl

Supporting information file. DOI: 10.1107/S2414314620007002/zl4041Isup3.cml

CCDC reference: 2005280

Additional supporting information:  crystallographic information; 3D view; checkCIF report

## Figures and Tables

**Table 1:** Hydrogen-bond geometry (Å, °)

*D*—H⋯*A*	*D*—H	H⋯*A*	*D*⋯*A*	*D*—H⋯*A*
C2—H2⋯S1^i^	0.93	3.00	3.699 (2)	133
C6—H6⋯F1^ii^	0.93	2.52	3.441 (3)	169

Symmetry codes: (i) 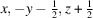 ; (ii) 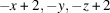 .

**Table 2:** Experimental details

Crystal data	
Chemical formula	C_25_H_18_ClF_2_N_5_S
*M*_r_	493.95
Crystal system, space group	Monoclinic, *P*2_1_/*c*
Temperature (K)	293
*a*, *b*, *c* (Å)	16.9376 (6), 13.1440 (4), 10.6399 (4)
β (°)	92.891 (4)
*V* (Å^3^)	2365.72 (14)
*Z*	4
Radiation type	Mo *K*α
μ (mm^−1^)	0.29
Crystal size (mm)	0.32 × 0.19 × 0.04

Data collection	
Diffractometer	Rigaku Oxford Diffraction SuperNova, Dual, Cu at zero, Atlas
Absorption correction	Gaussian (*CrysAlis PRO*; Rigaku OD, 2015 ▸)
*T*_min_, *T*_max_	0.727, 1.000
No. of measured, independent and observed [*I* > 2σ(*I*)] reflections	21848, 5930, 3549
*R*_int_	0.027
(sin θ/λ)_max_ (Å^−1^)	0.700

Refinement	
*R*[*F* ^2^ > 2σ(*F* ^2^)], *wR*(*F* ^2^), *S*	0.048, 0.125, 1.02
No. of reflections	5930
No. of parameters	364
No. of restraints	255
H-atom treatment	H-atom parameters constrained
Δρ_max_, Δρ_min_ (e Å^−3^)	0.14, −0.21

Computer programs: *CrysAlis PRO* (Rigaku OD, 2015 ▸), *SHELXS* (Sheldrick, 2008 ▸), *SHELXL* (Sheldrick, 2015 ▸), *CHEMDRAW Ultra* (Cambridge Soft, 2001 ▸) and *WinGX* (Farrugia, 2012 ▸).

## full crystallographic data

### Crystal data

**Table:**

C_25_H_18_ClF_2_N_5_S	*F*(000) = 1016
*M**_r_* = 493.95	*D*_x_ = 1.387 Mg m^−^^3^
Monoclinic, *P*2_1_/*c*	Mo *K*α radiation, λ = 0.71073 Å
*a* = 16.9376 (6) Å	Cell parameters from 6505 reflections
*b* = 13.1440 (4) Å	θ = 3.5–26.8°
*c* = 10.6399 (4) Å	µ = 0.29 mm^−^^1^
β = 92.891 (4)°	*T* = 293 K
*V* = 2365.72 (14) Å^3^	Plate, colourless
*Z* = 4	0.32 × 0.19 × 0.04 mm

### Data collection

**Table:**

Rigaku Oxford Diffraction SuperNova, Dual, Cu at zero, Atlas diffractometer	3549 reflections with *I* > 2σ(*I*)
ω scans	*R*_int_ = 0.027
Absorption correction: gaussian (CrysAlisPro; Rigaku OD, 2015)	θ_max_ = 29.9°, θ_min_ = 3.1°
*T*_min_ = 0.727, *T*_max_ = 1.000	*h* = −17→23
21848 measured reflections	*k* = −18→17
5930 independent reflections	*l* = −14→14

### Refinement

**Table:**

Refinement on *F*^2^	Primary atom site location: structure-invariant direct methods
Least-squares matrix: full	Secondary atom site location: difference Fourier map
*R*[*F*^2^ > 2σ(*F*^2^)] = 0.048	Hydrogen site location: inferred from neighbouring sites
*wR*(*F*^2^) = 0.125	H-atom parameters constrained
*S* = 1.02	*w* = 1/[σ^2^(*F*_o_^2^) + (0.0445*P*)^2^ + 0.6409*P*] where *P* = (*F*_o_^2^ + 2*F*_c_^2^)/3
5930 reflections	(Δ/σ)_max_ = 0.001
364 parameters	Δρ_max_ = 0.14 e Å^−^^3^
255 restraints	Δρ_min_ = −0.21 e Å^−^^3^

### Special details

**Table:**

Geometry. All esds (except the esd in the dihedral angle between two l.s. planes) are estimated using the full covariance matrix. The cell esds are taken into account individually in the estimation of esds in distances, angles and torsion angles; correlations between esds in cell parameters are only used when they are defined by crystal symmetry. An approximate (isotropic) treatment of cell esds is used for estimating esds involving l.s. planes.
Refinement. Non-hydrogen atoms were refined with anisotropic diaplacement parameters. All hydrogen atoms were placed in calculated positions and refined using a riding model. Difference Fourier maps showed that the methyl hydrogen atoms were disordered. The methyl group was therefore modelled as two components related by a 60° rotation about the C-C bond and the C-H bond distances were fixed at 0.96 Å, with displacement parameters 1.5 times Ueq(C). The hydrogen atoms were allowed to rotate freely and the occupancy ratio for the two components refined to 57 (3):43 (3)%. C-H distances for sp^2^ hybridized groups were set to 0.93Å and their U_iso_(H) set to 1.2 times the U_eq_(C). Methine and methylene C-H bond distances were fixed at 0.98 Å and 0.97 Å with displacement parameters 1.2 times Ueq(C).

### Fractional atomic coordinates and isotropic or equivalent isotropic displacement parameters (Å^2^)

**Table:**

	*x*	*y*	*z*	*U*_iso_*/*U*_eq_	Occ. (<1)
C1	0.91410 (14)	−0.11438 (18)	0.8797 (2)	0.0725 (6)	
C2	0.87189 (13)	−0.18539 (17)	0.8117 (2)	0.0713 (6)	
H2	0.855169	−0.245141	0.848925	0.086*	
C3	0.85468 (13)	−0.16585 (16)	0.6857 (2)	0.0671 (6)	
H3	0.826052	−0.213282	0.637229	0.081*	
C4	0.87930 (12)	−0.07710 (16)	0.6308 (2)	0.0621 (5)	
C5	0.92300 (13)	−0.00652 (17)	0.7036 (2)	0.0704 (6)	
H5	0.940622	0.053154	0.667359	0.085*	
C6	0.93986 (14)	−0.02567 (18)	0.8292 (2)	0.0774 (6)	
H6	0.968353	0.021092	0.878928	0.093*	
C7	0.85842 (12)	0.03407 (15)	0.3320 (2)	0.0626 (5)	
C8	0.87394 (12)	0.11841 (15)	0.2618 (2)	0.0656 (6)	
C9	0.80263 (12)	0.03318 (14)	0.1218 (2)	0.0601 (5)	
C10	0.92100 (14)	0.20868 (18)	0.3087 (3)	0.0885 (8)	
H10A	0.932863	0.201734	0.397511	0.133*	0.57 (3)
H10B	0.890877	0.269636	0.293062	0.133*	0.57 (3)
H10C	0.969369	0.212399	0.265659	0.133*	0.57 (3)
H10D	0.929210	0.254112	0.239977	0.133*	0.43 (3)
H10E	0.971196	0.186210	0.344426	0.133*	0.43 (3)
H10F	0.892703	0.243447	0.371829	0.133*	0.43 (3)
C11	0.74684 (12)	0.08582 (15)	−0.09277 (19)	0.0619 (5)	
H11	0.796880	0.108379	−0.125855	0.074*	
C12	0.70269 (14)	0.01744 (15)	−0.1895 (2)	0.0663 (6)	
H12A	0.652423	0.047161	−0.217543	0.080*	
H12B	0.733981	0.005413	−0.261916	0.080*	
C13	0.69096 (12)	−0.07930 (14)	−0.11686 (19)	0.0575 (5)	
C14	0.69975 (11)	0.17716 (14)	−0.05306 (19)	0.0558 (5)	0.767 (10)
C15	0.6495 (3)	0.1762 (4)	0.0439 (5)	0.0655 (13)	0.767 (10)
H15	0.643982	0.116965	0.090367	0.079*	0.767 (10)
C16	0.6071 (3)	0.2618 (4)	0.0739 (6)	0.0750 (14)	0.767 (10)
H16	0.573373	0.260671	0.140280	0.090*	0.767 (10)
C17	0.6154 (4)	0.3473 (4)	0.0048 (6)	0.0683 (12)	0.767 (10)
C18	0.6614 (2)	0.3505 (3)	−0.0949 (5)	0.0728 (11)	0.767 (10)
H18	0.664560	0.409543	−0.142531	0.087*	0.767 (10)
C19	0.7038 (2)	0.2650 (3)	−0.1254 (5)	0.0640 (10)	0.767 (10)
H19	0.735132	0.266155	−0.194617	0.077*	0.767 (10)
F2	0.5728 (5)	0.4315 (5)	0.0333 (6)	0.1030 (16)	0.767 (10)
C14B	0.69975 (11)	0.17716 (14)	−0.05306 (19)	0.0558 (5)	0.233 (10)
C15B	0.6368 (10)	0.1597 (14)	0.0220 (18)	0.062 (2)	0.233 (10)
H15B	0.624555	0.093644	0.045623	0.074*	0.233 (10)
C16B	0.5927 (11)	0.2392 (12)	0.061 (2)	0.066 (2)	0.233 (10)
H16B	0.549429	0.227761	0.109998	0.079*	0.233 (10)
C17B	0.6127 (14)	0.3346 (14)	0.029 (3)	0.072 (2)	0.233 (10)
C18B	0.6792 (8)	0.3578 (9)	−0.0333 (16)	0.070 (2)	0.233 (10)
H18B	0.693605	0.424663	−0.049414	0.084*	0.233 (10)
C19B	0.7238 (8)	0.2759 (8)	−0.0711 (16)	0.068 (2)	0.233 (10)
H19B	0.771021	0.287906	−0.109519	0.081*	0.233 (10)
F2B	0.5692 (13)	0.4152 (14)	0.0696 (19)	0.089 (4)	0.233 (10)
C20	0.64915 (12)	−0.16914 (15)	−0.16629 (18)	0.0575 (5)	
C21	0.60277 (15)	−0.16523 (18)	−0.2771 (2)	0.0760 (6)	
H21	0.596607	−0.103776	−0.319834	0.091*	
C22	0.56563 (16)	−0.2511 (2)	−0.3250 (2)	0.0843 (7)	
H22	0.535116	−0.247636	−0.400041	0.101*	
C23	0.57369 (13)	−0.34122 (17)	−0.2621 (2)	0.0705 (6)	
C24	0.61747 (14)	−0.34719 (17)	−0.1505 (2)	0.0766 (6)	
H24	0.621560	−0.408430	−0.106855	0.092*	
C25	0.65523 (13)	−0.26174 (16)	−0.1037 (2)	0.0688 (6)	
H25	0.685526	−0.265981	−0.028541	0.083*	
N1	0.85765 (10)	−0.06409 (13)	0.50112 (17)	0.0643 (4)	
N2	0.88125 (10)	0.01956 (14)	0.45499 (17)	0.0657 (5)	
N3	0.84226 (10)	0.11839 (12)	0.14170 (18)	0.0656 (5)	
N4	0.76260 (10)	0.01344 (12)	0.01117 (17)	0.0649 (5)	
N5	0.72506 (10)	−0.07906 (12)	−0.00653 (16)	0.0606 (4)	
S1	0.80048 (3)	−0.05346 (4)	0.24354 (5)	0.06286 (17)	
F1	0.93157 (10)	−0.13275 (12)	1.00396 (13)	0.1024 (5)	
Cl1	0.52834 (4)	−0.44993 (5)	−0.32473 (8)	0.1043 (3)	

### Atomic displacement parameters (Å^2^)

**Table:**

	*U*^11^	*U*^22^	*U*^33^	*U*^12^	*U*^13^	*U*^23^
C1	0.0781 (15)	0.0764 (15)	0.0627 (14)	−0.0006 (12)	0.0011 (11)	−0.0113 (12)
C2	0.0760 (14)	0.0675 (14)	0.0706 (14)	−0.0109 (11)	0.0055 (11)	−0.0077 (11)
C3	0.0659 (13)	0.0632 (13)	0.0721 (14)	−0.0078 (10)	0.0020 (11)	−0.0137 (11)
C4	0.0600 (12)	0.0591 (12)	0.0669 (13)	0.0036 (10)	0.0004 (10)	−0.0137 (10)
C5	0.0745 (14)	0.0599 (13)	0.0765 (15)	−0.0037 (11)	−0.0006 (12)	−0.0102 (11)
C6	0.0830 (16)	0.0709 (15)	0.0772 (16)	−0.0102 (12)	−0.0073 (12)	−0.0216 (12)
C7	0.0584 (12)	0.0511 (11)	0.0779 (14)	0.0062 (9)	0.0003 (10)	−0.0033 (10)
C8	0.0533 (12)	0.0491 (11)	0.0945 (17)	0.0045 (9)	0.0032 (11)	−0.0020 (11)
C9	0.0572 (12)	0.0472 (11)	0.0760 (14)	0.0085 (9)	0.0030 (10)	0.0070 (10)
C10	0.0753 (15)	0.0601 (13)	0.129 (2)	−0.0057 (12)	−0.0048 (15)	−0.0040 (14)
C11	0.0638 (12)	0.0512 (11)	0.0717 (13)	0.0044 (9)	0.0127 (10)	0.0152 (10)
C12	0.0831 (15)	0.0529 (11)	0.0639 (12)	0.0094 (11)	0.0130 (11)	0.0085 (10)
C13	0.0618 (12)	0.0502 (11)	0.0614 (12)	0.0103 (9)	0.0120 (10)	0.0059 (9)
C14	0.0557 (11)	0.0470 (10)	0.0648 (11)	0.0003 (8)	0.0053 (9)	0.0079 (9)
C15	0.079 (2)	0.056 (2)	0.063 (2)	0.0039 (17)	0.013 (2)	0.0137 (18)
C16	0.084 (3)	0.074 (3)	0.069 (2)	0.010 (2)	0.020 (2)	−0.003 (2)
C17	0.076 (2)	0.0528 (19)	0.075 (3)	0.0150 (17)	−0.0015 (19)	−0.0016 (17)
C18	0.089 (2)	0.0475 (15)	0.083 (3)	0.0061 (15)	0.007 (2)	0.0131 (17)
C19	0.069 (2)	0.0531 (16)	0.071 (2)	−0.0001 (14)	0.0117 (18)	0.0134 (16)
F2	0.130 (2)	0.0725 (19)	0.108 (4)	0.0422 (17)	0.018 (2)	−0.0035 (19)
C14B	0.0557 (11)	0.0470 (10)	0.0648 (11)	0.0003 (8)	0.0053 (9)	0.0079 (9)
C15B	0.068 (4)	0.051 (4)	0.066 (4)	−0.003 (4)	0.010 (4)	−0.002 (4)
C16B	0.072 (4)	0.058 (4)	0.067 (4)	−0.006 (4)	0.011 (4)	0.001 (4)
C17B	0.082 (4)	0.059 (4)	0.075 (5)	0.012 (4)	0.011 (4)	−0.005 (4)
C18B	0.082 (4)	0.050 (4)	0.080 (5)	0.005 (4)	0.010 (4)	0.006 (4)
C19B	0.070 (4)	0.055 (4)	0.078 (4)	0.000 (3)	0.015 (4)	0.007 (4)
F2B	0.100 (6)	0.078 (7)	0.087 (8)	0.024 (5)	0.005 (6)	−0.016 (6)
C20	0.0609 (12)	0.0531 (11)	0.0594 (12)	0.0084 (9)	0.0115 (9)	0.0009 (9)
C21	0.0975 (18)	0.0627 (14)	0.0674 (14)	0.0056 (12)	−0.0016 (13)	0.0049 (11)
C22	0.0931 (18)	0.0861 (18)	0.0726 (15)	0.0002 (14)	−0.0064 (13)	−0.0081 (14)
C23	0.0642 (13)	0.0632 (13)	0.0855 (16)	0.0021 (11)	0.0166 (12)	−0.0146 (12)
C24	0.0768 (15)	0.0545 (13)	0.0985 (18)	0.0049 (11)	0.0053 (14)	0.0052 (12)
C25	0.0711 (14)	0.0565 (12)	0.0780 (14)	0.0037 (10)	−0.0028 (11)	0.0074 (11)
N1	0.0658 (11)	0.0576 (10)	0.0690 (11)	0.0032 (8)	−0.0017 (9)	−0.0066 (9)
N2	0.0623 (11)	0.0583 (10)	0.0761 (12)	0.0066 (8)	0.0001 (9)	−0.0066 (9)
N3	0.0594 (10)	0.0475 (9)	0.0895 (13)	0.0030 (8)	0.0009 (9)	0.0075 (9)
N4	0.0748 (11)	0.0442 (9)	0.0750 (12)	0.0024 (8)	−0.0038 (9)	0.0108 (8)
N5	0.0678 (11)	0.0465 (9)	0.0675 (11)	0.0032 (8)	0.0030 (9)	0.0073 (8)
S1	0.0725 (3)	0.0461 (3)	0.0696 (3)	0.0013 (2)	0.0004 (3)	0.0034 (2)
F1	0.1286 (13)	0.1099 (11)	0.0673 (9)	−0.0177 (9)	−0.0094 (8)	−0.0066 (8)
Cl1	0.0951 (5)	0.0835 (5)	0.1349 (6)	−0.0123 (4)	0.0109 (4)	−0.0357 (4)

### Geometric parameters (Å, º)

**Table:**

C1—C2	1.361 (3)	C14—C15	1.370 (5)
C1—F1	1.362 (3)	C14—C19	1.391 (4)
C1—C6	1.364 (3)	C15—C16	1.381 (5)
C2—C3	1.381 (3)	C15—H15	0.9300
C2—H2	0.9300	C16—C17	1.354 (6)
C3—C4	1.379 (3)	C16—H16	0.9300
C3—H3	0.9300	C17—C18	1.348 (5)
C4—C5	1.397 (3)	C17—F2	1.364 (5)
C4—N1	1.420 (3)	C18—C19	1.380 (4)
C5—C6	1.375 (3)	C18—H18	0.9300
C5—H5	0.9300	C19—H19	0.9300
C6—H6	0.9300	C14B—C19B	1.376 (10)
C7—N2	1.359 (3)	C14B—C15B	1.383 (13)
C7—C8	1.370 (3)	C15B—C16B	1.363 (13)
C7—S1	1.755 (2)	C15B—H15B	0.9300
C8—N3	1.361 (3)	C16B—C17B	1.348 (13)
C8—C10	1.501 (3)	C16B—H16B	0.9300
C9—N3	1.317 (2)	C17B—C18B	1.371 (14)
C9—N4	1.354 (3)	C17B—F2B	1.371 (13)
C9—S1	1.726 (2)	C18B—C19B	1.386 (11)
C10—H10A	0.9600	C18B—H18B	0.9300
C10—H10B	0.9600	C19B—H19B	0.9300
C10—H10C	0.9600	C20—C21	1.385 (3)
C10—H10D	0.9600	C20—C25	1.389 (3)
C10—H10E	0.9600	C21—C22	1.377 (3)
C10—H10F	0.9600	C21—H21	0.9300
C11—N4	1.473 (2)	C22—C23	1.364 (3)
C11—C14B	1.513 (3)	C22—H22	0.9300
C11—C14	1.513 (3)	C23—C24	1.370 (3)
C11—C12	1.532 (3)	C23—Cl1	1.739 (2)
C11—H11	0.9800	C24—C25	1.373 (3)
C12—C13	1.506 (3)	C24—H24	0.9300
C12—H12A	0.9700	C25—H25	0.9300
C12—H12B	0.9700	N1—N2	1.276 (2)
C13—N5	1.282 (2)	N4—N5	1.380 (2)
C13—C20	1.461 (3)		
			
C2—C1—F1	118.5 (2)	C20—C13—C12	124.86 (18)
C2—C1—C6	123.2 (2)	C15—C14—C19	118.3 (3)
F1—C1—C6	118.4 (2)	C15—C14—C11	124.0 (3)
C1—C2—C3	117.9 (2)	C19—C14—C11	117.6 (2)
C1—C2—H2	121.1	C14—C15—C16	121.2 (4)
C3—C2—H2	121.1	C14—C15—H15	119.4
C4—C3—C2	121.0 (2)	C16—C15—H15	119.4
C4—C3—H3	119.5	C17—C16—C15	118.7 (5)
C2—C3—H3	119.5	C17—C16—H16	120.7
C3—C4—C5	119.3 (2)	C15—C16—H16	120.7
C3—C4—N1	116.42 (18)	C18—C17—C16	122.2 (4)
C5—C4—N1	124.2 (2)	C18—C17—F2	118.8 (4)
C6—C5—C4	119.7 (2)	C16—C17—F2	118.8 (4)
C6—C5—H5	120.1	C17—C18—C19	119.1 (3)
C4—C5—H5	120.1	C17—C18—H18	120.4
C1—C6—C5	118.9 (2)	C19—C18—H18	120.4
C1—C6—H6	120.5	C18—C19—C14	120.3 (3)
C5—C6—H6	120.5	C18—C19—H19	119.8
N2—C7—C8	125.8 (2)	C14—C19—H19	119.8
N2—C7—S1	123.29 (16)	C19B—C14B—C15B	118.6 (9)
C8—C7—S1	110.82 (17)	C19B—C14B—C11	123.0 (5)
N3—C8—C7	115.76 (19)	C15B—C14B—C11	117.5 (8)
N3—C8—C10	119.3 (2)	C16B—C15B—C14B	120.1 (13)
C7—C8—C10	124.9 (2)	C16B—C15B—H15B	120.0
N3—C9—N4	122.10 (19)	C14B—C15B—H15B	120.0
N3—C9—S1	118.06 (17)	C17B—C16B—C15B	119.1 (15)
N4—C9—S1	119.83 (15)	C17B—C16B—H16B	120.4
C8—C10—H10A	109.5	C15B—C16B—H16B	120.4
C8—C10—H10B	109.5	C16B—C17B—C18B	123.6 (13)
H10A—C10—H10B	109.5	C16B—C17B—F2B	119.5 (14)
C8—C10—H10C	109.5	C18B—C17B—F2B	116.6 (15)
H10A—C10—H10C	109.5	C17B—C18B—C19B	116.2 (11)
H10B—C10—H10C	109.5	C17B—C18B—H18B	121.9
C8—C10—H10D	109.5	C19B—C18B—H18B	121.9
H10A—C10—H10D	141.1	C14B—C19B—C18B	121.5 (9)
H10B—C10—H10D	56.3	C14B—C19B—H19B	119.2
H10C—C10—H10D	56.3	C18B—C19B—H19B	119.2
C8—C10—H10E	109.5	C21—C20—C25	117.8 (2)
H10A—C10—H10E	56.3	C21—C20—C13	121.36 (18)
H10B—C10—H10E	141.1	C25—C20—C13	120.86 (19)
H10C—C10—H10E	56.3	C22—C21—C20	120.9 (2)
H10D—C10—H10E	109.5	C22—C21—H21	119.6
C8—C10—H10F	109.5	C20—C21—H21	119.6
H10A—C10—H10F	56.3	C23—C22—C21	119.8 (2)
H10B—C10—H10F	56.3	C23—C22—H22	120.1
H10C—C10—H10F	141.1	C21—C22—H22	120.1
H10D—C10—H10F	109.5	C22—C23—C24	120.8 (2)
H10E—C10—H10F	109.5	C22—C23—Cl1	119.6 (2)
N4—C11—C14B	112.39 (17)	C24—C23—Cl1	119.62 (19)
N4—C11—C14	112.39 (17)	C23—C24—C25	119.3 (2)
N4—C11—C12	100.91 (15)	C23—C24—H24	120.4
C14B—C11—C12	113.99 (17)	C25—C24—H24	120.4
C14—C11—C12	113.99 (17)	C24—C25—C20	121.4 (2)
N4—C11—H11	109.7	C24—C25—H25	119.3
C14—C11—H11	109.7	C20—C25—H25	119.3
C12—C11—H11	109.7	N2—N1—C4	114.00 (17)
C13—C12—C11	102.89 (16)	N1—N2—C7	114.31 (18)
C13—C12—H12A	111.2	C9—N3—C8	108.94 (17)
C11—C12—H12A	111.2	C9—N4—N5	119.63 (16)
C13—C12—H12B	111.2	C9—N4—C11	126.37 (17)
C11—C12—H12B	111.2	N5—N4—C11	113.68 (16)
H12A—C12—H12B	109.1	C13—N5—N4	108.07 (16)
N5—C13—C20	121.30 (18)	C9—S1—C7	86.43 (10)
N5—C13—C12	113.75 (18)		

### Hydrogen-bond geometry (Å, º)

**Table:**

*D*—H···*A*	*D*—H	H···*A*	*D*···*A*	*D*—H···*A*
C2—H2···S1^i^	0.93	3.00	3.699 (2)	133
C6—H6···F1^ii^	0.93	2.52	3.441 (3)	169

Symmetry codes: (i) *x*, −*y*−1/2, *z*+1/2; (ii) −*x*+2, −*y*, −*z*+2.
